# The novel object-matching test (NOM Test): A psychometric measure of visual comparison ability

**DOI:** 10.3758/s13428-023-02069-6

**Published:** 2023-02-14

**Authors:** Bethany Growns, Alice Towler, Kristy Martire

**Affiliations:** 1 School of Psychology, Speech and Hearing, University of Canterbury, Christchurch, New Zealand; 2 School of Social and Behavioural Sciences, Arizona State University, Tempe, AZ USA; 3 School of Psychology, University of New South Wales, Kensington, Australia; 4 School of Psychology, The University of Queensland, Brisbane, Australia

**Keywords:** Object-matching, Visual comparison, Perceptual expertise, Forensics, Face-matching

## Abstract

This paper presents a new test of object-matching ability: the Novel Object-Matching Test (NOM Test). Object-matching (or visual comparison) is a complex cognitive and perceptual visual comparison task undertaken by forensic scientists – yet no openly available, standardised and psychometrically validated test of object-matching ability exists. This is in contrast to other visual comparison domains like face-matching where many tests are widely available. In this paper, we present the development and psychometric validation of the first openly available object-matching test where people view two complex artificial visual patterns side-by-side and decide if they are from the same source or different sources. We provide normative data and psychometric properties for two long-form and two short-form versions of the test, and two additional versions designed to identify high and low-performers. We also provide evidence of discriminant validity and convergent validity that demonstrates the NOM Test correlates strongly with other object-matching tasks like fingerprint-matching – but not other tasks requiring cognitive-perceptual skill (e.g., visual intelligence). The NOM Test is free for research use with acknowledgment and is available at https://osf.io/pv6ye/.

Visual comparison is a complex cognitive and perceptual task where visual stimuli are compared by eye. One important real-world visual comparison domain is forensic visual comparison – sometimes also referred to as pattern-matching or object-matching – where viewers compare visual objects side-by-side to decide if they are from the same source or different sources (Busey & Dror, 2011; Towler et al., 2018; *visual comparison* and *object-matching* are henceforth used interchangeably). For example, whether two fingerprints were made by the same finger or two different fingers, or whether the striations on two firearms casings were made by the same gun or two different guns (see Fig. 1). Object-matching is a stable cognitive ability that generalises across multiple types of visual patterns – from fingerprints to firearms (Growns et al., 2022a). However, no standardized, psychometrically validated tests of object-matching ability currently exist. Reliable measurement of this skill is critical to understanding the cognitive and perceptual processes that determine performance on this task. It is also critical to empirically investigating how errors in this task can be reduced – particularly where erroneous forensic visual comparison judgements can result in the wrongful conviction of innocent people (Garrett & Neufeld, 2009).

**Fig. 1:**
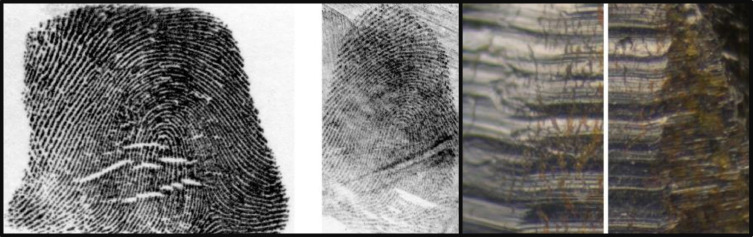
Are these patterns from the same source or different source? These fingerprints (*left panel*) were taken from the same person, and these digitised firearms cartridges (*right panel*) were fired from the same gun

The majority of research investigating visual comparison ability focuses solely on face-matching. Many studies have demonstrated that there are reliable individual differences in face-matching (Fox & Bindemann, 2020; McCaffery et al., 2018; Wilmer, 2017) and several reliable tests of this ability already exist (e.g., Burton et al., 2010; Dunn et al., 2020; Fysh & Bindemann, 2018; Stantic et al., 2022; White et al., 2021). Yet studies investigating *object-*matching largely investigate group-level differences (e.g., performance differences between novices and forensic science examiners; Busey & Vanderkolk, 2005; Thompson & Tangen, 2014) – with few investigating individual differences in this ability (although see Richler et al., 2019 for an exception in object *recognition*). Research has only recently identified reliable and stable individual differences in object-matching (Growns et al., 2022a; Searston & Tangen, 2017). Yet few reliable and psychometrically validated tests of object-matching ability are available. This may be partially due to privacy and security concerns making visual pattern stimuli like fingerprints publicly available, but also the difficulty forensic laboratories face in sharing stimuli across jurisdictions and the feasibility of developing large databases of real-world pattern stimuli.

Nevertheless, it is critical that reliable standardised tests of visual comparison ability are available and made openly accessible for research use. This will be vital to investigating the potential overlap or independence of face-matching and object-matching ability. For example, it may be the case that these skills are largely dissociable because faces and other visual stimuli are processed in different neural regions (Grill-Spector et al., 2004; Kanwisher et al., 1997), because humans are already considered ‘experts’ in face processing (but not other visual stimuli; Young & Burton, 2018), or because of the different task demands of face and object-matching (i.e., face-matching requires comparison across non-rigid variations like expression and pose which object-matching does not require; White et al., 2021). Yet it is difficult to investigate such questions without being able to test both face and more general object-matching abilities.

Face-matching research has also identified ‘super-recognisers’ who perform well above the norm in both face-matching and recognition (Noyes et al., 2017; Russell et al., 2009), as well as individuals with prosopagnosia or significant impairment in these abilities (Shah et al., 2015; White et al., 2017). It is possible that individuals with object-matching skill – or ‘super-matchers’ – also exist, or even individuals with clinical impairment in this skill. Yet we are also unable to answer these questions without the availability of both object-matching and face-matching tests.

There are also important applied reasons that reliable measures of object-matching ability are needed. In the legal system, forensic examiners complete forensic visual comparison to link or exclude suspects from crime scenes – for example, comparing a suspect’s fingerprint with a crime scene fingerprint. Thousands of these comparisons are made by forensic examiners around the world each day (Thompson et al., 2013). A reliable object-matching test could be used in practice to identify and recruit ‘super-matchers’ to improve professional performance and reduce costly real-world errors – similar to the way ‘super-recognisers’ are being recruited in police and security settings (Robertson et al., 2016). Reliable object-matching tests would also enable research investigating the relationships between object-matching and other cognitive processes, such as visual search (Robson et al., 2021), statistical learning (Growns et al., 2022b; Growns & Martire, 2020a) and non-analytical processing (Thompson & Tangen, 2014). A comprehensive understanding of the cognitive mechanisms implicated in object-matching could inform the development of training programs to also further reduce real-world errors (Growns & Martire, 2020b).

## A novel psychometric measure of object-matching performance

In this paper, we present the development and validation of a new measure of visual comparison performance: the Novel Object-Matching Test (NOM Test). We adapt the test development approach used by White et al. (2021) to develop and present several versions of the NOM Test established using psychometric principles. We also provide normative data, discriminant, and convergent validity for these tests.

The NOM Test has several advantages over existing visual comparison tests: a) we provide two calibrated versions of long and short NOM Test to reliably assess object-matching performance across time, as well as two versions that are specifically calibrated to identify high and low performers; b) it is composed of entirely novel stimuli so can be used as a measurement of object-matching skill for both professionals and novices; and c) is openly available for research use and avoids potential privacy concerns associated with sharing other forensically relevant pattern stimuli (e.g., fingerprints).

Below we present the development and validation of the following versions of the NOM Test:

- The 160-item NOM Test Long Version comprising two equally difficult 80-item test forms (NOM-A and NOM-B). These are not intended to be the primary measure of object-matching but are a starting point for selecting comparisons with the most desirable psychometric properties.
- The 80-item NOM Test Short Version (NOM-S) comprising two equally difficult 40-item test forms (NOM-SA and NOM-SB). These versions will be useful in future longitudinal or intervention research.
- The 40-item NOM Test-High is designed to discriminate high-performing individuals that are likely to be useful in research with professionals and other top-performers.
- The 40-item NOM Test-Low is designed to discriminate low-performing individuals. This version of the test is designed to be an inverse of the NOM Test-High and could be helpful in research investigating populations with clinical impairment in visual perceptual abilities (e.g., prosopagnosia).

These tests are free for research use with appropriate acknowledgement. The materials, data, and the pre-registration of the three-phase test validation are available on OSF (https://osf.io/pv6ye/). The materials are accompanied by procedural documentation and normative data to assist future research.

We report object-matching performance throughout this paper using both raw accuracy and a signal detection measure of sensitivity (d'; Phillips et al., 2001; Stanislaw & Todorov, 1999). Statistical analyses for test–retest reliability, discriminant validity, and convergent validity were conducted using d' to minimise any patterns of response bias towards responding ‘same’ or ‘different’ in our results (White et al., 2021). Psychometric analyses were conducted using raw data accuracy where necessary and all analyses are also supplemented with descriptive raw accuracy statistics. 

## Test construction and development

### Test item creation

**Fig. 2:**
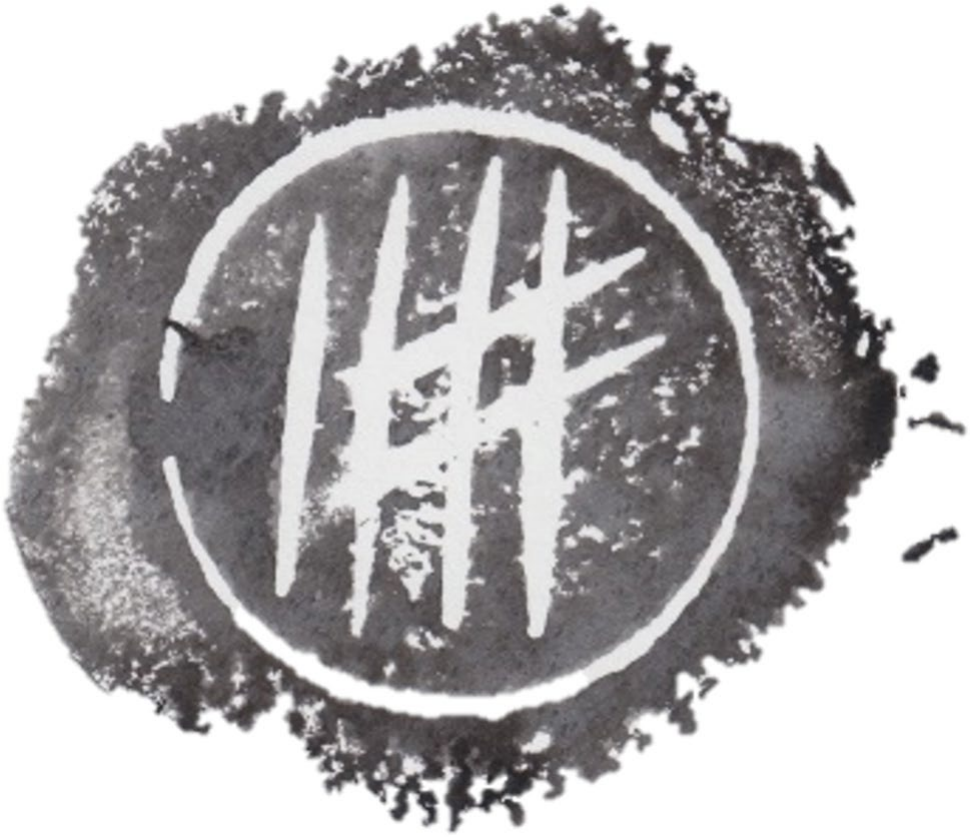
An example of a 'raw' stamp impression after it was halved, carved, stamped, dried, scanned, and digitised (prior to cropping)

The NOM Test was developed using a database of stamp impressions developed by the authors. The database comprises 312 stamp impressions created by halving 26 potatoes, pressing a circular ring into each cross section to create a uniform circular outline, and then carving three vertical lines intersected by two lines within that boundary using a single carving tool (see Fig. 2). Each impression was then dipped in black ink and pressed onto cardboard six times to create six stamp impressions of the 52 potato-halves. Each stamp impression was then digitised by scanning each print at 600 pdi and then cropped around the circular boundary so that the silhouette of the potato did not act as a cue to the source. This process resulted in 312 artificial-prints (six stamp impressions from each of 52 potato-halves).

**Fig. 3:**
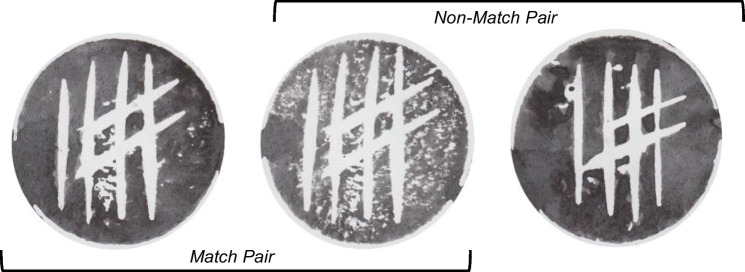
Examples of a match and non-match pairs for trials where participants were asked to answer the question ‘Are these prints from the same stamping tool or different stamping tools?’

Pilot studies were conducted to first identify the most dissimilar matching pair and non-matching pairs. To create the *match* pairs, we assigned the first impression from each potato-half as the target print (*n* = 52) and presented pilot participants with the subsequent five stamp impressions from the same potato in an array. Participants were asked to choose which of the prints in the array was most dissimilar to the target. *Non-match* pairs were created by randomly pairing the 52 target prints with a randomly selected non-match foil from all non-match foils (without replacement).^1^ Example trials can be seen in Fig. 3. This process of trial construction resulted in the first version of the test consisting of 104 artificial-print trials (52 match and 52 non-match). This version of the test has been used in recent research investigating individual differences in object-matching performance (e.g., Growns et al., 2022a).

We then created an additional 208 trials from the remaining match and non-match foils. Match trials were created by randomly pairing matched foils with the constraints: 1) that no foils from the first 104 trials were repeated and 2) no foils that were stamped immediately after one another in the foil generation sequence (i.e., impressions 2–6) were paired together to reduce the number of highly similar match pairs. The non-match trials were created by randomly pairing non-match foils with the constraint that none of the foils from the first 104 trials were repeated for match and non-match pairs.

### Phase one: Item difficulty screening

To develop long and short versions of the NOM Test, we first conducted an initial phase of data collection that involved screening the 312 test items for difficulty. We recruited 300 participants from Prolific Academic (*N* = 293 after seven exclusions based on our pre-registered exclusion criteria; M_age_ = 38.3 SD = 13.7; 144 males, 141 females, and eight gender-diverse; all located in the US) who were compensated US $3.34 for participation in an approximately 25-min study. Participants were randomly assigned to one of six randomly generated versions of the test comprising 52 trials presented in a random order (26 match, 26 non-match).

To assist participants in the task, we informed them that the prints were created using a specific art tool that was dipped in ink and then stamped on paper to create decorative patterns. We told them that the art tools were hand-carved and so each tool leaves a unique stamp. Participants were informed that they would be shown two prints at a time and were asked to decide if they were from the same art tool or different tools (see Materials on OSF for full instructions). On each trial, participants viewed two potato-prints side-by-side and were asked ‘are these prints from the same stamping tool or two different stamping tools?’ They responded by selecting one of two buttons (‘same’ or ‘different’) at the bottom of the screen.

Participants in the first phase of data collection achieved an average accuracy of 75.6% (SD = 9.3; M = 75.6%, SD = 16.3 on match trials; M = 75.5%, SD = 17.1 on non-match trials), demonstrating that the match and non-match trials were equally difficult. We used the item screening data to select trials for the NOM Test – Long version (*n* = 160 trials) comprised of two equally difficult forms of the test (*n* = 80 trials each, 40 match and 40 non-match trials; Long Form A: M = 75.3%, SD = 17.1, Long Form B: M = 75.3%, SD = 16.6). We selected trials by calculating the item-to-test correlation (i.e., how well accuracy on each trial predicts each participant’s overall performance) for each trial in the item screening data. This method identifies trials that are most predictive of overall test performance and provides an overall estimate of a trial’s contribution to test reliability (Guilford, 1954; Wilmer et al., 2012). Item-to-test correlations were Pearson’s correlations between each participant’s response on each trial (0 = *incorrect*, 1 = *correct*) and the same participants’ d-prime for all other trials. This was done to select the trials that were most predictive of performance whilst avoiding any influence response bias (i.e., tendency to respond 'same' or 'different'; Stanislaw & Todorov, 1999) on trial selection (White et al., 2021). The average item-to-test correlations were substantially above zero for each version of the test (Long Form A: M = 0.34, SD = 0.09; Long Form B: M = 0.35, SD = 0.10).

### Phase two: Normative scores, test reliability of long-form tests, convergent and discriminant validity

We then recruited a new group of participants to collect normative data, test–retest reliability, and measures of convergent and discriminant validity for both long-form versions of the NOM Test. Participants completed two versions of the NOM Test in two sessions approximately 1 week apart, and also completed measures of convergent and discriminant validity in the first session (described further below).

We recruited 299 participants from Prolific Academic (M_age_ = 40.0, SD = 13.1; 148 males, 146 females, five gender-diverse; all located in the US) to complete the first session. Participants were compensated US $6.40 for a 45-min study. Participants (*N* = 279) who completed the second session were compensated $2.70 for a 20-min study and an additional $1.00 bonus for completing both sessions. The order of tests was counterbalanced so that half of participants completed Long Form A first and Long Form B second, and vice versa for the other half of participants. Participants completed all trials in each session in a randomised order.

#### Normative data and psychometric properties

We first established normative data for both versions of the test using the data from the first data collection session. Performance on Long Form Version A was 76.2% (SD = 0.10, *min* = 0.46, *max* = 0.94) and 76.7% (SD = 0.11, *min* = 0.46, *max* = 0.95) on Long Form Version B. Accuracy did not significantly differ between the two tests in the first data collection session (Long Form A d' M = 1.66, SD = 0.70, Long Form B d' M = 1.71, SD = 0.76; *t*_(294.87)_ = 0.61, *p* = 0.545). Both versions of the test were normally distributed as kurtosis scores were close to 3 (Long Form A: 3.11, Long Form B: 2.80), and were both moderately negatively skewed (Long Form A: –0.68, Long Form B: –0.64). These distributions and negative skew are comparable to psychometrically validated tests of face-matching (e.g., GFMT2; White et al., 2021).

#### Test–retest reliability

**Fig. 4:**
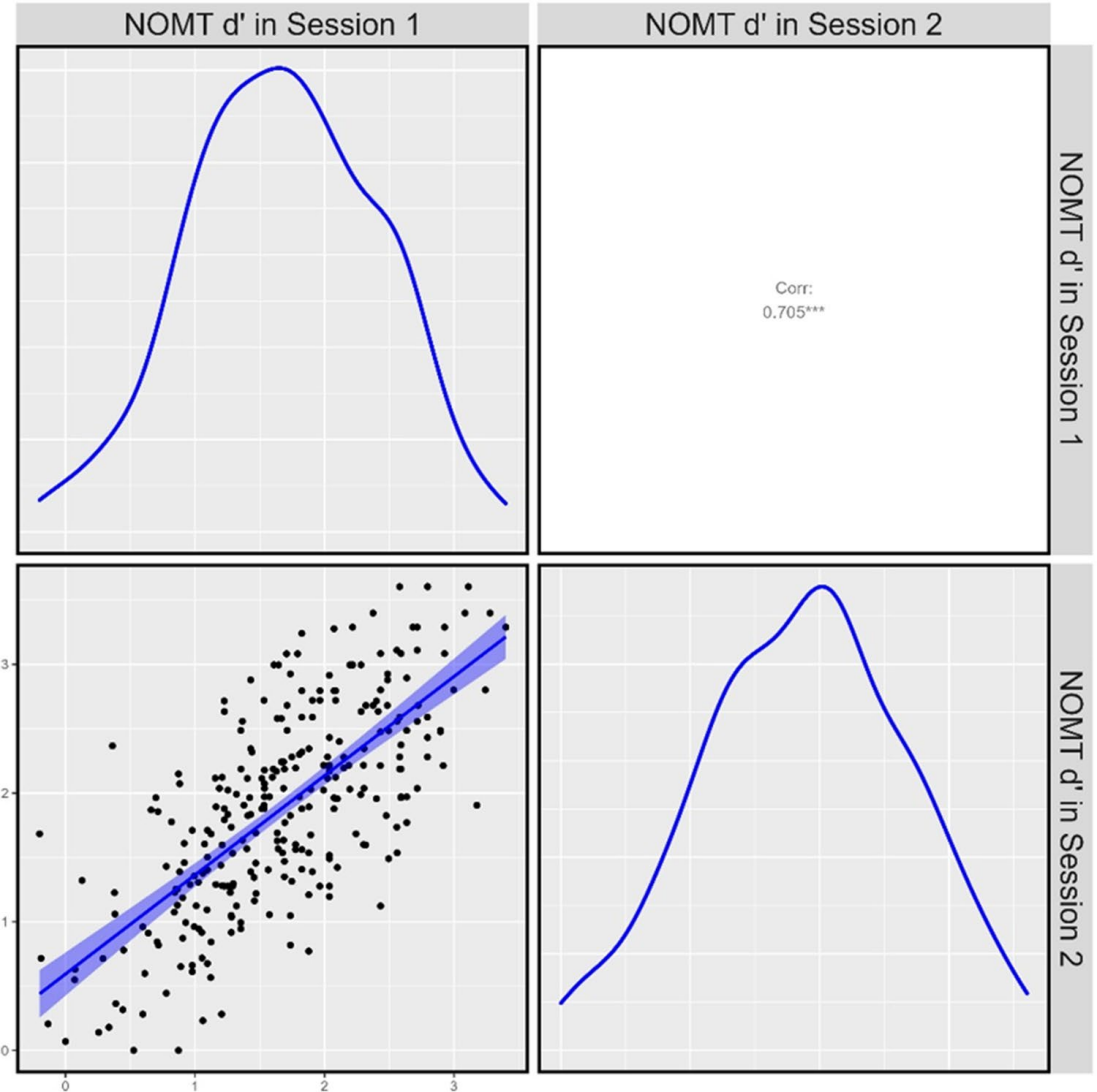
Correlation between average sensitivity (d') on the NOM Test during first and second data collection sessions in Phase Two

The correlation and distributions of accuracy in the first and second data collection sessions is illustrated in Fig. 4. Test–retest reliability was high (*r*_(277)_ = 0.705, *p* < 0.001) and comparable to existing tests of face-matching (e.g., *r* = 0.68 in Murray & Bate, 2020; *r* = 0.78 in White et al., 2021; and *r* = 0.70 in Wilmer et al., 2010). Internal test reliability calculated using data from all participants in the first data collection session was also high for both long form versions of the test (A: *n* = 150, Cronbach’s α = 0.83; B: *n* = 149, α = 0.83).

#### Convergent validity

Participants (*n* = 299) completed fingerprint-matching and face-matching tasks as measures of convergent validity during Session 1. Participants completed a fingerprint-matching test to establish convergent validity that the NOM Test measures object-matching ability. They completed a face-matching test to provide further evidence that the NOM Test is a better measure of object-matching – than face-matching – ability by investigating whether the performance on the NOM Test is better predicted by face or fingerprint-matching ability (e.g., as seen in Growns et al., 2022a).

Participants completed two different versions of fingerprint-matching and face-matching tests to increase the generalisability of the results: they completed one of two versions of the Fingerprint-Matching Test (FMT) from Growns, Towler, et al. (2022c) and completed either the GFMT-S (Burton et al., 2010) or GFMT2-S (White et al., 2021). Participants who completed NOM-SA completed the first FMT in Growns, Dunn, et al. (2022a); Growns, Mattijssen, et al. (2022b); Growns, Towler, et al. (2022c) and the GFMT2-S (White et al., 2021). Participants who completed the NOM-SB completed the second FMT in Growns, Towler, et al. (2022c) and the GFMT-S (Burton et al., 2010). Data from both fingerprint-matching and both face-matching tests were pooled for convergent validity correlational analyses.

**Fig. 5:**
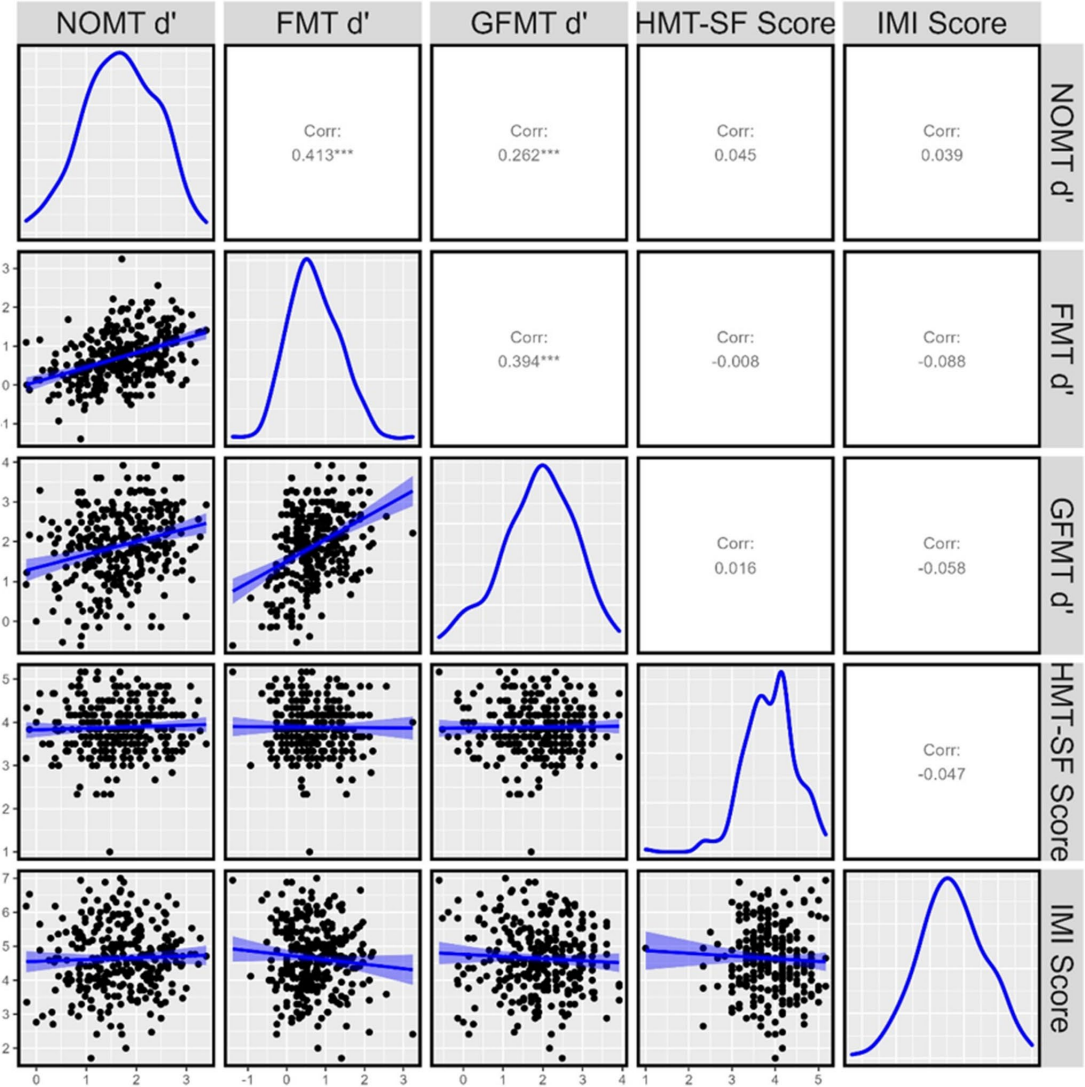
Correlation between average sensitivity (d') on the NOM Test [NOMT] during Session 1 in Phase 2 and measures of convergent (Fingerprint-Matching Test [FMT] and Glasgow Face-Matching Test [GFMT]) and divergent (Hagen Matrices Test – Short Form [HMT-SF] and the Intrinsic Motivation Inventory [IMI]) validity

The NOM Test significantly correlated with both fingerprint and face-matching accuracy (see Fig. 5) – providing evidence of high convergent validity and suggesting that the NOM Test taps into a broader object-matching ability. These results – combined with additional research demonstrating that the NOM Test positively correlates with other pattern-matching tests like firearms (Growns et al., 2022a) – suggest that the NOM Test is a reliable test for object-matching performance. The correlation observed between the NOM Test and fingerprint-matching (*r* = .41) is also within a similar range seen between standardised measures of face-matching (Dunn et al., 2020; McCaffery et al., 2018).

And consistent with the hypothesis that the NOM Test is more strongly associated with object-matching ability, the NOM Test had a significantly stronger association with fingerprint-matching performance (*r* = 0.41, *p* < 0.001), than face-matching (*r* = 0.26, *p* < 0.001; *t* = 2.58, *p* < 0.010). This pattern of results is also consistent with previous research demonstrating face-matching had the lowest average correlation with several other object-matching tests (Growns et al., 2022a). These results suggests that the NOM Test is a better test of object-matching than face-matching. Yet the existence of a small but significant correlation between face-matching and object-matching does provides a hint that the two abilities may be related – but further research will be needed to investigate their overlap or independence.

#### Discriminant validity

Participants also completed two measures of discriminant validity during Session 1: the Hagen Matrices Test – Short Form and the Intrinsic Motivation Inventory. The Hagen Matrices Test – Short Form (HMT-SF; Heydasch et al., 2013) was used to confirm that the NOM Test measures object-matching ability rather than general visual intelligence ability. The HMT-SF is a measure of visual reasoning and intelligence and is comprised of six trials where participants are instructed to look for patterns in 3 × 3 visual matrices, identify the underlying rules, and select a correct answer to complete each matrix within 2 min. HFMT-SF scores were calculated by averaging participants’ total correct and incorrect responses. The Intrinsic Motivation Inventory (IMI; McAuley et al., 1989) was used to confirm that the NOM Test measures object-matching ability rather than participants’ motivation and enjoyment of the task. The IMI is a measure of intrinsic motivation and is composed of eighteen questions (e.g., ‘I put a lot of effort into this.’) answered on seven-point Likert scales from ‘Not At All True’ to ‘Very True.’ IMI scores were calculated by averaging participants’ Likert-scale responses where higher scores indicate higher intrinsic motivation.

The NOM Test did not significantly correlate with either the HMT-SF or the IMI (see Fig. 5) – providing evidence of discriminant validity and suggesting that the NOM Test taps into object-matching ability, rather than visual intelligence or intrinsic motivation. It is also consistent with other research showing that artificial-print-matching performance isn’t significantly associated with other abilities that require visual-perceptual skill, including visual search (Chan & Hayward, 2013; Ericson et al., 2017) and visual statistical learning ability (Growns et al., 2020; Growns et al., 2022a; Siegelman & Frost, 2015).

### Phase three: Development, normative scores, and test reliability of short-form tests

We next created three short versions of the test to provide more efficient and diverse test options. The primary version of the short test consists of two 40-trial tasks calibrated to be of equal difficulty to enable repeat testing (NOM-SA and SB). Trials in the NOM-SA and SB were selected by calculating the item-to-test correlation for each trial in both long-form versions of the test using data from Session 1 in the previous phase of data collection. We computed item-to-test correlations for each trial, ranked the items by item-test correlation, selected the 40 match and 40 non-match trials with the highest correlations and then created two versions of the test of approximately equal difficulty (difficulty was equated for both overall accuracy and accuracy separate on match and non-match trials).

Two additional versions of the test were created to identify top-performers (NOM-High) and low-performers (NOM-Low). The NOM-High was created by selecting 40 trials with the highest item-to-test correlations for top-performing participants (i.e., above the median) in the first session of data collection in phase two. The NOM-Low was likewise created by selecting 40 trials with the highest item-to-test correlations for low-performing participants (i.e., below the median).

To provide normative scores and test–retest reliability for the NOM Test – Short version (NOM-S), we recruited another 302 participants. Participants completed two versions of the test approximately 1 week apart and the order of tests was counterbalanced so that half of participants completed Short Form A first and Short Form B second, and vice versa for the other half of participants. Participants completed trials in a set order to minimise error variance (Mollon et al., 2017) – something that is also recommended in face-matching tasks (White et al., 2021). We recommend using a fixed order for all versions of the NOM Test.

**Fig. 6:**
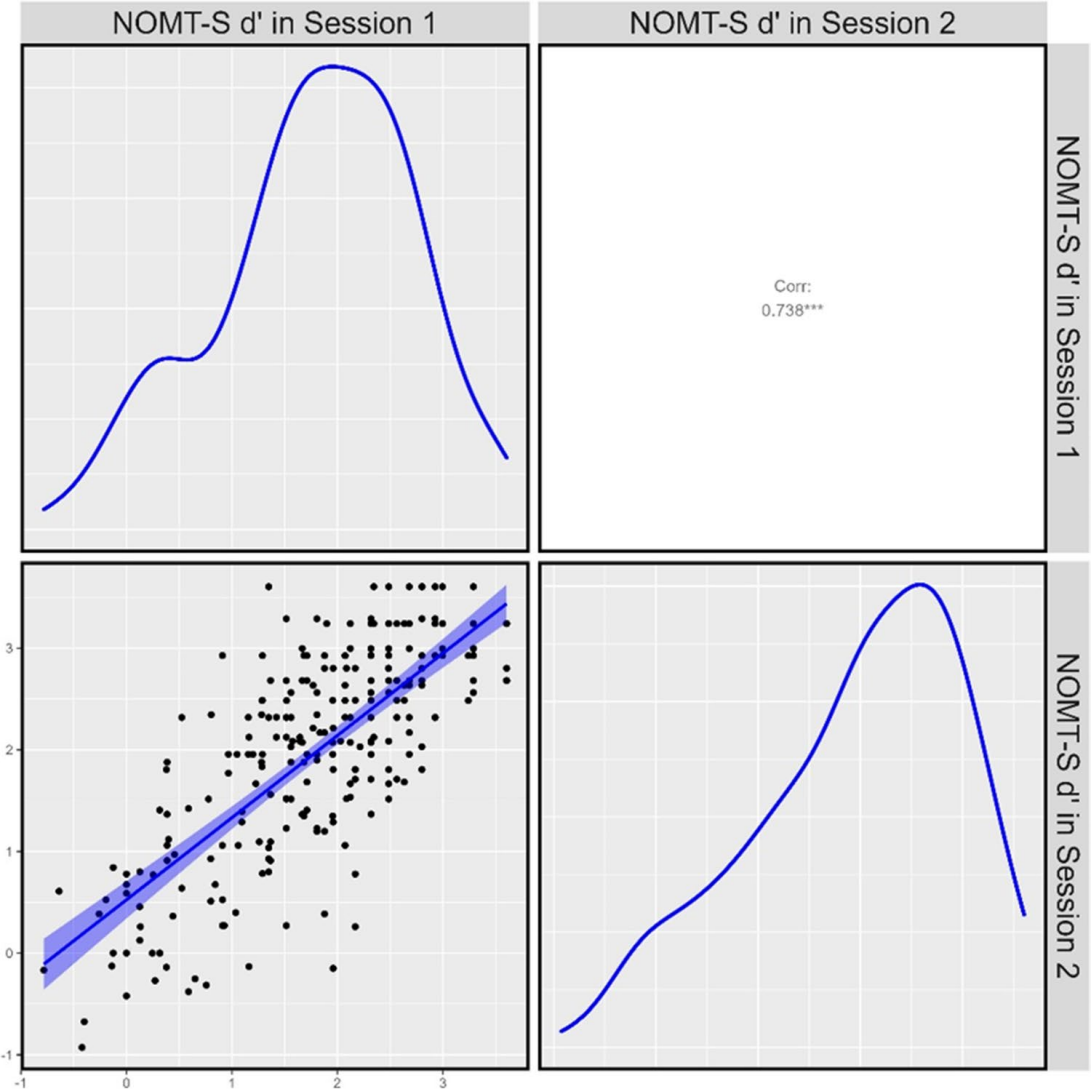
Correlation between average sensitivity (d') on the NOM-S Test during first and second data collection sessions in phase three

To increase the generalisability of our findings, we recruited participants from both the United States and the United Kingdom (US *N* = 150, UK *N* = 152). Accuracy did not significantly differ between these populations during Session 1 (US d' M = 1.72, SD = 0.96, UK d' M = 1.89, SD = 0.92; *t*_(299.13)_ = 1.58, *p* = 0.115), or for those who completed Session 2 (US *N* = 130, d' M = 1.85, SD = 1.06; UK *N* = 125 d' M = 2.08, SD = 0.98; *t*_(252.71)_ = 1.77, *p* = 0.079). We thus combined data across these populations to for all results reported below. Normative accuracy from the first data collection session on the NOM-SA was 76.4% (SD = 13.8) and NOM-SB was 79.1% (SD = 12.7) and test–retest reliability was high (*r* = 0.740; see Fig. 6).

We also evaluated the psychometric properties of both short versions of the test using data from the first data collection session. Both versions of the test were normally distributed as kurtosis scores were close to 3 (Short Form A: 3.44, Long Form B: 2.92), and were both moderately negatively skewed (Short Form A: -0.90, Short Form B: -0.83). Internal test reliability calculated using data from all participants in the first data collection session was also high for both short-form versions of the test (SA: *n* = 151, Cronbach’s α = 0.79; SB: *n* = 151, α = 0.83). These psychometric properties are comparable with psychometrically validated tests of face-matching (e.g., GFMT2; White et al., 2021).

## Discussion

In this paper, we present the first publicly available test of visual comparison (or object-matching) – the Novel Object-Matching Test (NOM Test), along with normative data and psychometric properties of the task. We developed six versions of the test for use in various research contexts: two Long Form and two Short Form versions of the test for use in experimental and longitudinal studies, and two Short Form versions of the test designed for high and low performers to facilitate research on top-performers and those with potential clinical impairment in this ability. All six versions of the NOM Test had stable psychometric properties and stable test–retest reliability – providing a reliable standardised test for use in individual differences and group research. We also provided further evidence that the NOM Test taps into broader object-matching skill better than face-matching ability (see also Growns, Dunn, et al., 2022a).

The vast majority of matching tests available only measure face-matching ability. The NOM Test provides a new measure that can test broader visual comparison performance. This is critical to developing research investigating individual differences in object-matching performance, and research investigating the overlap or independence of object-matching and face-matching. It also provides a novel test to facilitate critical research investigating how to improve real-world performance in forensic feature-comparison disciplines. Super-recognisers identified via face-matching tests have begun to be recruited by organisations like the London Metropolitan Police to improve professional face-matching performance (Robertson et al., 2016). The NOM Test could inspire research investigating the potential presence of ‘super-matchers’ in the general population that could be similarly recruited by forensic laboratories.

## Data Availability

All studies in this paper were pre-registered and the data and analysis scripts can be found on the Open Science Framework: https://osf.io/pv6ye/

## Footnotes

1 We chose this approach because an earlier pilot study pairing target prints with their most similar non-match foil created unfeasibly difficult trials.
