# Correction to: The novel object-matching test (NOM Test): A psychometric measure of visual comparison ability

**DOI:** 10.3758/s13428-023-02154-w

**Published:** 2023-06-20

**Authors:** Bethany Growns, Alice Towler, Kristy Martire

**Affiliations:** 1 School of Psychology, Speech and Hearing, University of Canterbury, Christchurch, New Zealand; 2 School of Social and Behavioural Sciences, Arizona State University, Tempe, AZ USA; 3 School of Psychology, University of New South Wales, Kensington, Australia; 4 School of Psychology, The University of Queensland, Brisbane, Australia

**Correction to: Behavior Research Methods**

10.3758/s13428-023-02069-6

**Abstract**

This erratum reports on a technical error in the second phase of data collection in Growns et al. (2023). Due to this technical error, one finding about the relationship between the Novel Object-Matching Test (NOM test) and a measure of divergent validity (Hagen Matrices Test – Short Form) reported in the original paper was erroneous. We briefly describe the error and report the re-analyzed results accounting for the error. These re-analyzed findings do not change the overall conclusions or psychometric properties reported about the NOM test in the original paper and provide further insight into the cognitive mechanisms that may underpin visual comparison (also referred to as object-matching).

**The original analysis and technical error**

In the second phase of data collection in Growns et al. (Growns et al., 2023), we collected data from 299 participants from Prolific Academic to collect normative data, test–retest reliability, and measures of convergent and divergent validity for the Novel Object-Matching Test (NOM test). Participants completed two test sessions approximately 1 week apart and participants in the first testing session completed the following measures: the NOM Test – Long Form (A or B), the Fingerprint-Matching Test from (FMT; Growns et al., 2022), the Glasgow Face-Matching Test (GFMTS; Burton et al., 2010; or GFMT2-S White et al., 2021), the Hagen Matrices Test – Short Form (HMT-SF; Heydasch et al., 2013), and the Intrinsic Motivation Inventory (IMI; McAuley et al., 1989). The HMT-SF and IMI were included in the test battery as divergent validity measures of fluid visual intelligence and intrinsic motivation, respectively.

The technical error in the original paper was the reported HMT-SF scores that were calculated in the analysis code by averaging raw responses of all six trials – not the sum of the total correct scores as in Heydasch et al. (2013). This resulted in an error in analysis where we reported no significant relationship between the NOM Test – Long Form and HMT-SF (*r* = .045).

**Reanalysis of phase two data**

We re-analyzed the data investigating the relationship between HMT-SF scores (correctly calculated as the sum of correct scores) and all other measures in the second test session in the second phase of data collection (see Fig. 1). In the reanalysis, HMT-SF scores significantly correlated with sensitivity on the NOM test (*r* = .423). These re-analyzed results indicate that visual fluid intelligence and pattern completion skill (i.e., HMT-SF scores) is a convergent (rather than divergent) measure of visual comparison.

**Fig. 1:**
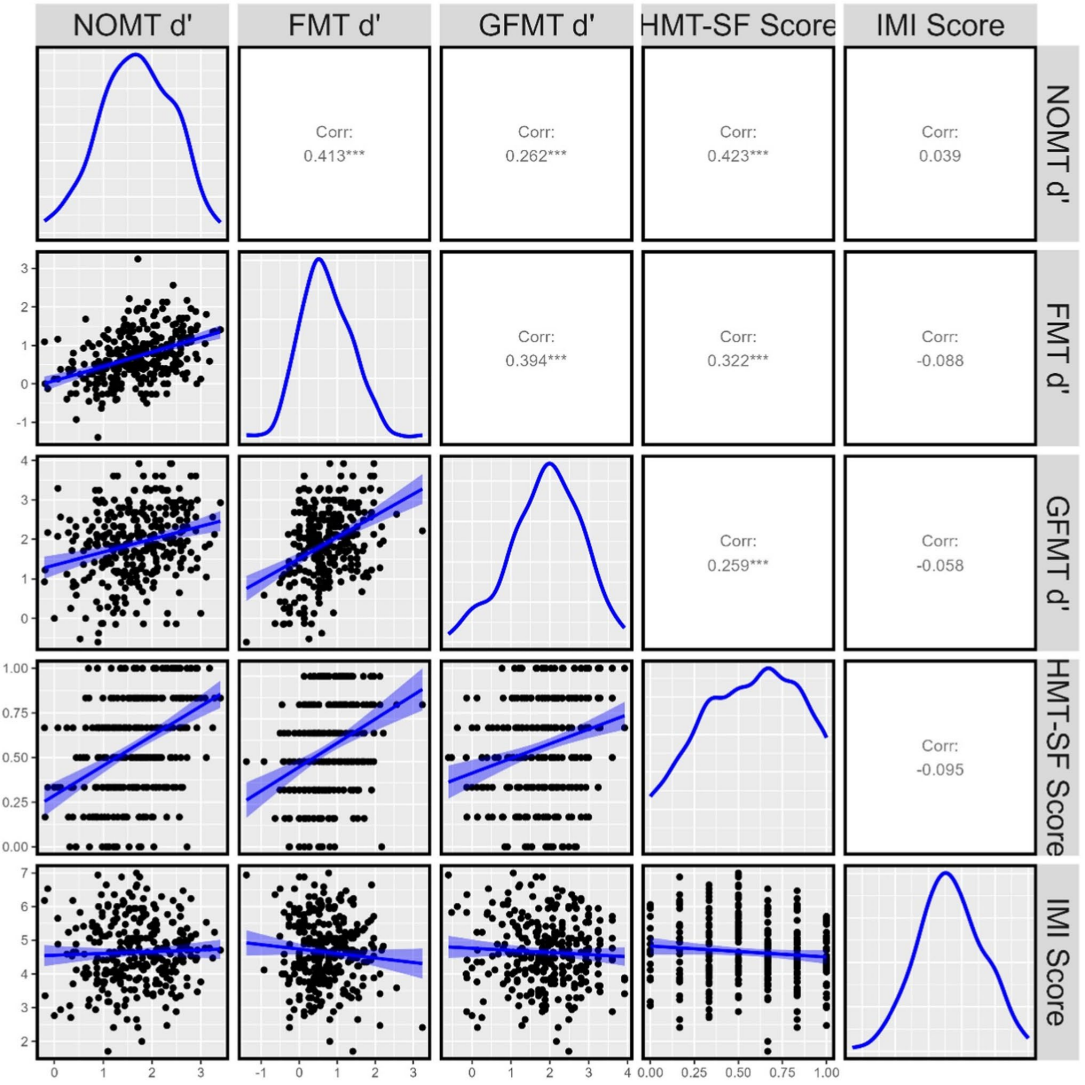
Correlation between average sensitivity (d') on the NOM Test [NOMT] during Session 1 in Phase 2 and measures of convergent (Fingerprint-Matching Test [FMT] and Glasgow Face-Matching Test [GFMT]) and divergent (Hagen Matrices Test – Short Form [HMT-SF] and the Intrinsic Motivation Inventory [IMI]) validity

**Conclusion**

We report the results of a reanalysis of data from Growns et al. (2023) that show an association between fluid visual intelligence (i.e., HMT-SF scores) and visual comparison (NOM test). Although we originally included the HMT-SF as a measure of divergent validity, this reanalysis reveals that fluid visual intelligence is actually a convergent measure of object-matching. This suggests that fluid visual intelligence is another cognitive mechanism that underlies object-matching ability. This is consistent with other research that demonstrates a moderate but significant relationship between fluid intelligence and object recognition (Richler et al., 2019).

This reanalysis provides further evidence of the key conclusions reported in the original paper: the NOM test taps into a broader object-matching and pattern completion ability (rather than face-matching skill) and is a reliable measure of object-matching. Importantly, the NOM test does not correlate with other divergent validity measures, such as intrinsic motivation or other tasks requiring cognitive-perceptual skill (i.e., visual search or visual statistical learning; Growns, Dunn, et al., 2022) – suggesting that object-matching is distinct to these psychological processes. Future research should continue to investigate the cognitive mechanisms that underpin visual comparison to further test how it can be reliably measured.

